# Attitudes and Perceptions of Canadian Otolaryngology‐Head and Neck Surgeons and Residents on Environmental Sustainability

**DOI:** 10.1002/oto2.40

**Published:** 2023-02-23

**Authors:** Kalpesh Hathi, James Fowler, Sarah Zahabi, Agnieszka Dzioba, Edward Madou, Anna C. Gunz, Leigh J. Sowerby, Anthony C. Nichols, Julie E. Strychowsky

**Affiliations:** ^1^ Faculty of Medicine Dalhousie Medicine New Brunswick Saint John New Brunswick Canada; ^2^ Department of Otolaryngology–Head and Neck Surgery Western University Ontario London Canada; ^3^ Department of Paediatrics Western University Ontario London Canada

**Keywords:** carbon savings, climate change, environmental sustainability, global warming, operating room, otolaryngology, recycling

## Abstract

**Objective:**

Healthcare systems, specifically operating rooms, significantly contribute to greenhouse gas emissions. Addressing operating room environmental sustainability requires understanding current practices, opinions, and barriers. This is the first study assessing the attitudes and perceptions of otolaryngologists on environmental sustainability.

**Study Design:**

Cross‐sectional virtual survey.

**Setting:**

Email survey to active members of the Canadian Society of Otolaryngology–Head and Neck Surgery.

**Methods:**

A 23‐question survey was developed in REDCap. The questions focused on four themes: (1) demographics, (2) attitudes and beliefs, (3) institutional practices, and (4) education. A combination of multiple choice, Likert‐scale, and open‐ended questions were employed.

**Results:**

Response rate was 11% (n = 80/699). Most respondents strongly believed in climate change (86%). Only 20% strongly agree that operating rooms contribute to the climate crisis. Most agree environmental sustainability is very important at home (62%) and in their community (64%), only 46% said it was very important in the operating room. Barriers to environmental sustainability were incentives (68%), hospital supports (60%), information/knowledge (59%), cost (58%), and time (50%). Of those involved in residency programs, 89% (n = 49/55) reported there was no education on environmental sustainability or they were unsure if there was.

**Conclusion:**

Canadian otolaryngologists strongly believe in climate change, but there is more ambivalence regarding operating rooms as a significant contributor. There is a need for further education and a systemic reduction of barriers to facilitate eco‐action in otolaryngology operating rooms.

Climate change is recognized to be the most significant threat to global health in the 21st century.^1^, ^2^, ^3^ Canada's greenhouse gas (GHG) emissions have increased by 13.1% from 1990 to 2020.^3^ However, Canada has joined numerous countries in pledging to reach net‐zero carbon emissions by 2050 and has signed a United Nations agreement to decarbonize the healthcare sector.^4^, ^5^, ^6^ It has been estimated that Canada's healthcare system generates 33 million tons of GHG emissions annually, making it responsible for 4.6% of the national total and the third leading GHG industry emitter.^7^, ^8^ This issue seems consistent internationally, as healthcare in the United States contributes 8.5% of the nation's GHG emissions.^9^ Globally, healthcare is estimated to contribute 4.4% of total emissions, which means that if healthcare was an country, it would be the 5th largest global emitter.^10^

Operating rooms (ORs) are responsible for a large portion of a hospital's environmental footprint. ORs use 3 to 6 times more energy than the hospital's average unit and produce 20% to 33% of total hospital waste.^11^, ^12^, ^13^ Previous studies have shown that up to 30% of OR solid waste is inappropriately labelled as infectious waste.^14^, ^15^ Assessments of Otolaryngology–Head and Neck Surgery (OHNS) ORs have found that up to 23.1% of the generated waste was recyclable.^16^, ^17^ Interventions including increasing reusable materials/gowns/equipment and improving the labeling of products and waste bins in the OR can reduce the environmental impacts.^18^, ^19^, ^20^, ^21^, ^22^

Previous surveys regarding OR environmental sustainability have been conducted among anesthesiologists.^18^, ^23^, ^24^, ^25^ However, only the Meyer et al study has involved surgeons, and this was limited to 2 academic hospitals in the United States.^26^ These surveys reported that most respondents wanted to improve environmental sustainability.^18^, ^23^, ^24^, ^25^, ^26^ However, they faced barriers including lack of support, lack of education, lack of resources, and negative attitudes.^18^, ^23^, ^24^, ^25^, ^26^ To address environmental sustainability in OHNS ORs, we need to understand current practices, opinions, and barriers.

This study assesses the attitudes, perceptions, practice patterns, and educational experiences of Canadian OHNS staff, residents, and fellows on environmental sustainability. This is the first study on this topic within OHNS.

## Methods

### 1 Study Design

This virtual cross‐sectional survey was approved from Western University's Health Sciences Research Ethics Board, Project ID: 121114, Informed consent was obtained from survey participants on the first page of the survey portal.

### 2 Survey Development

The survey was developed through internal discussion of the study authors and by previous surveys on environmental sustainability within healthcare.^18^, ^23^, ^24^, ^25^, ^26^ The survey questions focused on four themes: (1) demographics, (2) attitudes and beliefs, (3) institutional practices, and (4) education.

Prior to distribution, the survey was piloted on 2 OHNS residents and 2 attendings. The survey was revised using their feedback. The final survey was developed in REDCap (Research Electronic Data Capture) and consisted of 23‐questions representing a combination of multiple‐choice, Likert scale, and open‐ended questions. Open‐ended questions asked participants to describe any sustainability initiatives that have been conducted as well as any barriers, future directions, or personal feelings regarding environmental sustainability. Identical versions of the survey were available in English and French (Supplemental Material 1 and 2).

### 3 Survey Dissemination

The Canadian Society of Otolaryngology‐Head and Neck Surgery (CSOHNS) Electronics Media Editor approved the survey. It was then distributed to all 699 active members of the CSOHNS (195 residents/fellows, 504 staff). A reminder email was sent after 4 weeks. The survey was closed 3 weeks after this reminder email, allowing a total of 7 weeks for survey completion.

### 4 Data Analysis

Quantitative survey responses were analyzed using the Statistical Package for Social Sciences version 26 (SPSS 26) and Microsoft Excel 2010. Quantitative survey responses were described with absolute counts and percentages. For questions about environmental beliefs/attitudes, the responses from residents/fellows were compared to staff responses using a *χ* ^2^ test to assess for a generational difference. An *α* level of <.05 was set a‐priori.

Qualitative survey responses underwent exploratory content analysis. An inductive approach was used to analyze open‐ended question responses following the process of preparation (immersion and review of text data), organizing (coding and categorization), and reporting (conceptualization and description of categories).^27^

## Results

### 1 Response Rate

The survey was disseminated to all 699 active members of the CSOHNS on September 11, 2022, with a reminder on October 12, 2022. Eighty responses were received, yielding a response rate of 11.4%. Fourteen residents/fellows responded, representing 17.5% of the responses. This yields a resident/fellow response rate of 7.2% (n = 14/195). Sixty‐six attendings responded, representing 82.5% of total responses. This yields an attending response rate of 13.1% (n = 66/504).

### 2 Demographics

Demographic data are summarized in Table 1. The majority of attendings had been in practice ≤19 years (65.2%), and general otolaryngology was the most represented practice type (51.5%). Most respondents were 30 to 49 years old (61.3%), identified as male (71.3%), and practiced in an academic setting (58.8%). The province with the most responses was Ontario (42.5%).

**Table 1:** Respondent Demographics.

Variable	Overall, n = 80 (%)	Attending, n = 66 (82.5%)	Resident/fellow, n = 14 (17.5%)
Practice type			
General		34 (51.5)	
Pediatric		7 (10.6)	
Rhinology		10 (15.2)	
Laryngology		2 (3.0)	
Otology		12 (18.2)	
Head and neck oncology		5 (7.6)	
Facial plastic surgery		2 (3.0)	
Sleep surgery		0	
Years practicing			
<10		25 (37.9)	
10‐19		18 (27.3)	
20‐29		10 (15.2)	
30+		13 (19.7)	
Age			
20‐29	7 (8.8)	1 (1.5)	6 (42.9)
30‐39	27 (33.8)	19 (28.8)	8 (57.1)
40‐49	22 (27.5)	22 (33.3)	0
50‐59	16 (20.0)	16 (24.2)	0
60‐69	7 (8.8)	7 (10.6)	0
70+	1 (1.3)	1 (1.5)	0
Gender			
Male	57 (71.3)	47 (71.2)	10 (71.4)
Female	22 (27.5)	18 (27.3)	4 (28.6)
Not specified	1 (1.3)	1 (1.5)	0
Province of practice			
Alberta	11 (13.8)	9 (13.6)	2 (14.3)
British Columbia	14 (17.5)	13 (19.7)	1 (7.1)
Manitoba	2 (2.5)	1 (1.5)	1 (7.1)
New Brunswick	3 (3.8)	3 (4.5)	0
Newfoundland and Labrador	3 (3.8)	3 (4.5)	0
Nova Scotia	6 (7.5)	5 (7.6)	1 (7.1)
Ontario	34 (42.5)	25 (37.9)	9 (64.3)
Quebec	5 (6.3)	5 (7.6)	0
Saskatchewan	1 (1.3)	1 (1.5)	0
Not specified	1 (1.3)	1 (1.5)	0
Practice setting			
Isolated/remote	1 (1.3)	1 (1.5)	0
Rural	5 (6.3)	5 (7.6)	0
Urban	27 (33.8)	26 (39.4)	1 (7.1)
Academic	47 (58.8)	34 (51.5)	13 (92.9)

### 3 Attitudes and Beliefs

**Figure 1:**
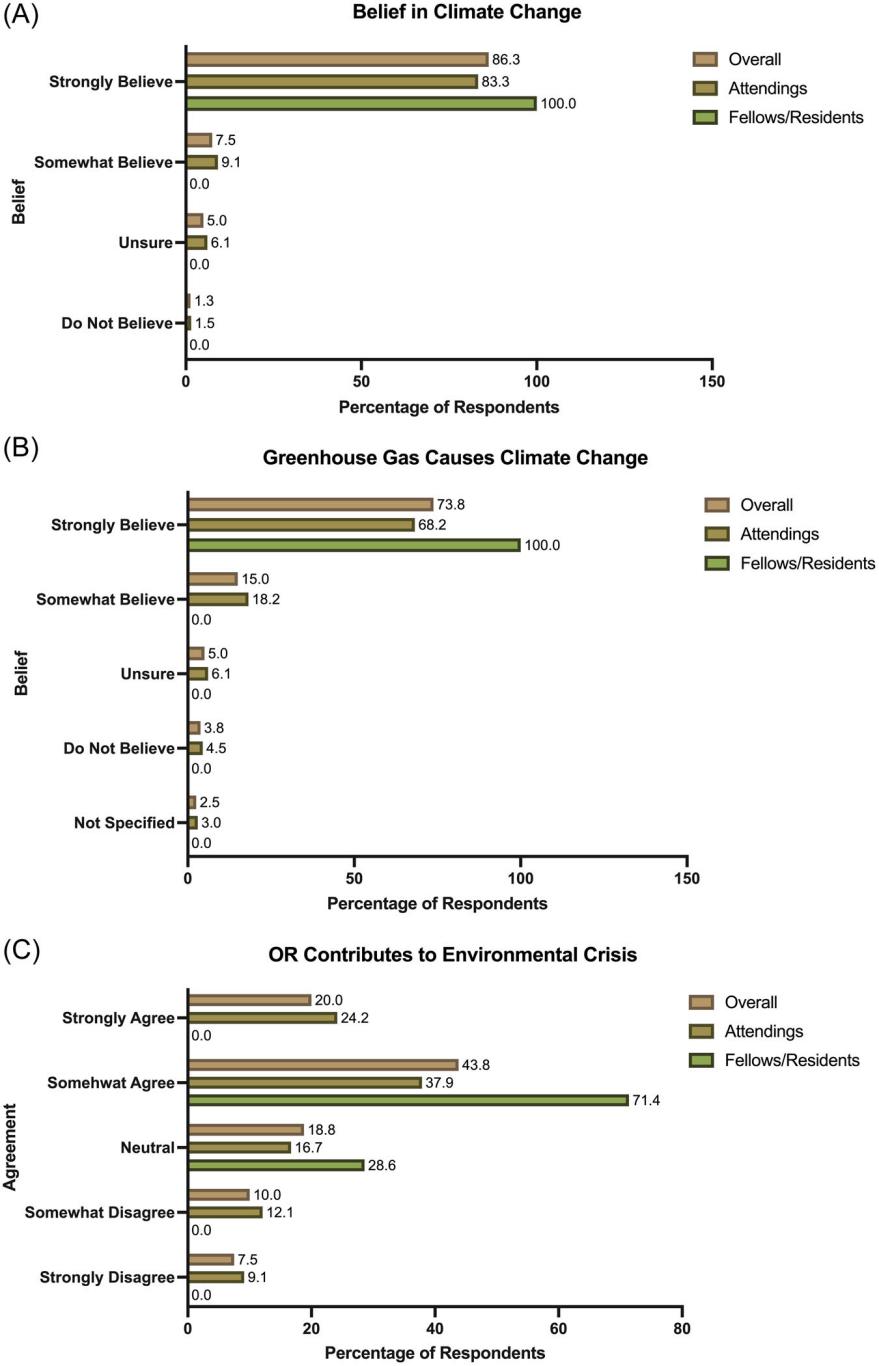
(A‐C). Respondents' beliefs toward climate change were reported for all respondents, attendings only, and fellows/residents only. OR, operating room.

Most respondents strongly believed climate change is occurring (86%) and GHGs cause climate change (74%) (Figure 1A‐C). There was no significant difference between attendings' and residents'/fellows' responses. However, only 20% of respondents strongly agree that ORs contribute to the environmental crisis, and 31% somewhat agree with this statement (Figure 1A‐C). There was a significant difference in the level of agreement between attendings and residents/fellows on this statement (*p* = .037) (Figure 1C).

**Figure 2:**
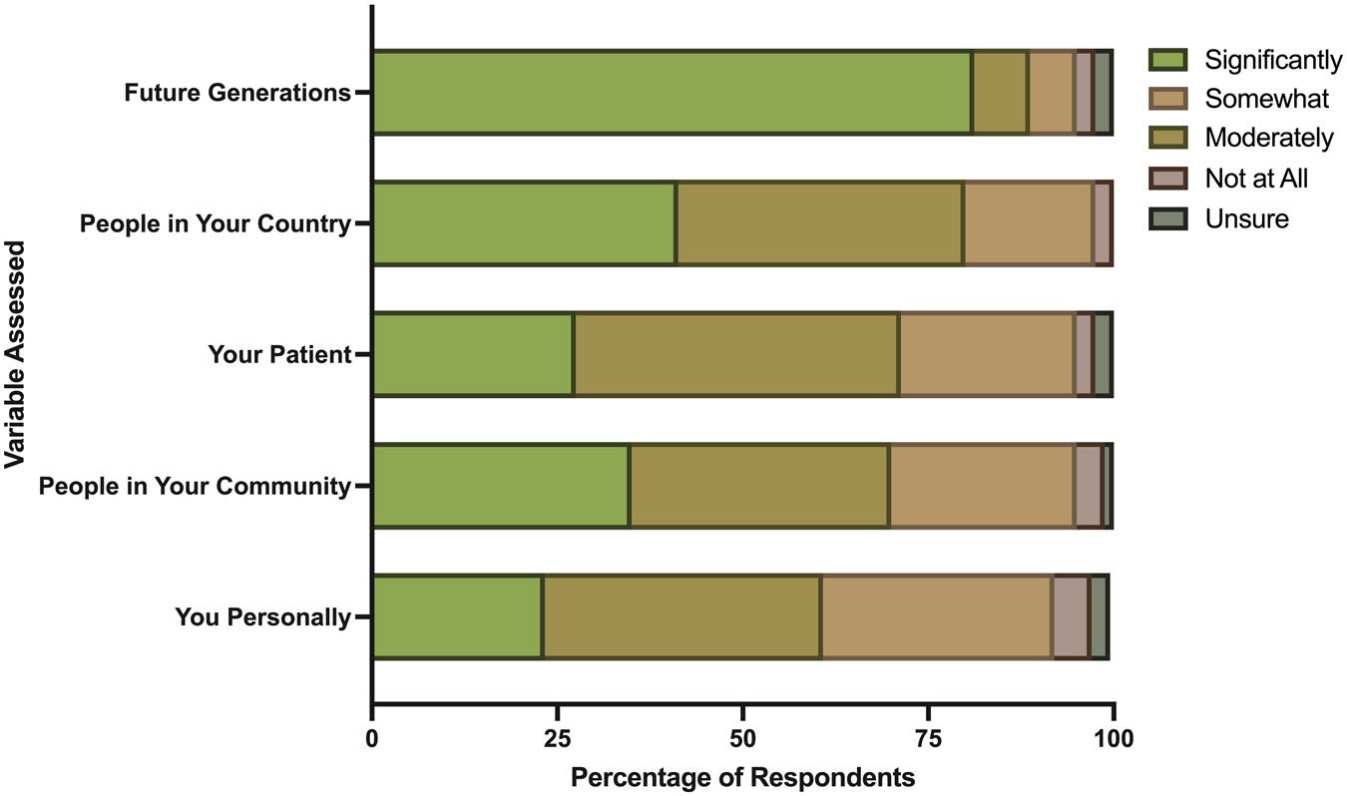
Belief that climate change will impact the following aspects of the respondents' lives.

Most respondents believed climate change will significantly harm future generations (81%) (Figure 2). However, there was less agreement as to whether climate change will significantly harm them personally (23%), people in their community (35%), their patients (28%), and people in their country (41%) (Figure 2). Respondents still felt that climate change would moderately or somewhat impact their communities, patients, and people in their country (Figure 2). There was no significant difference between attendings' and residents'/fellows' responses.

**Figure 3:**
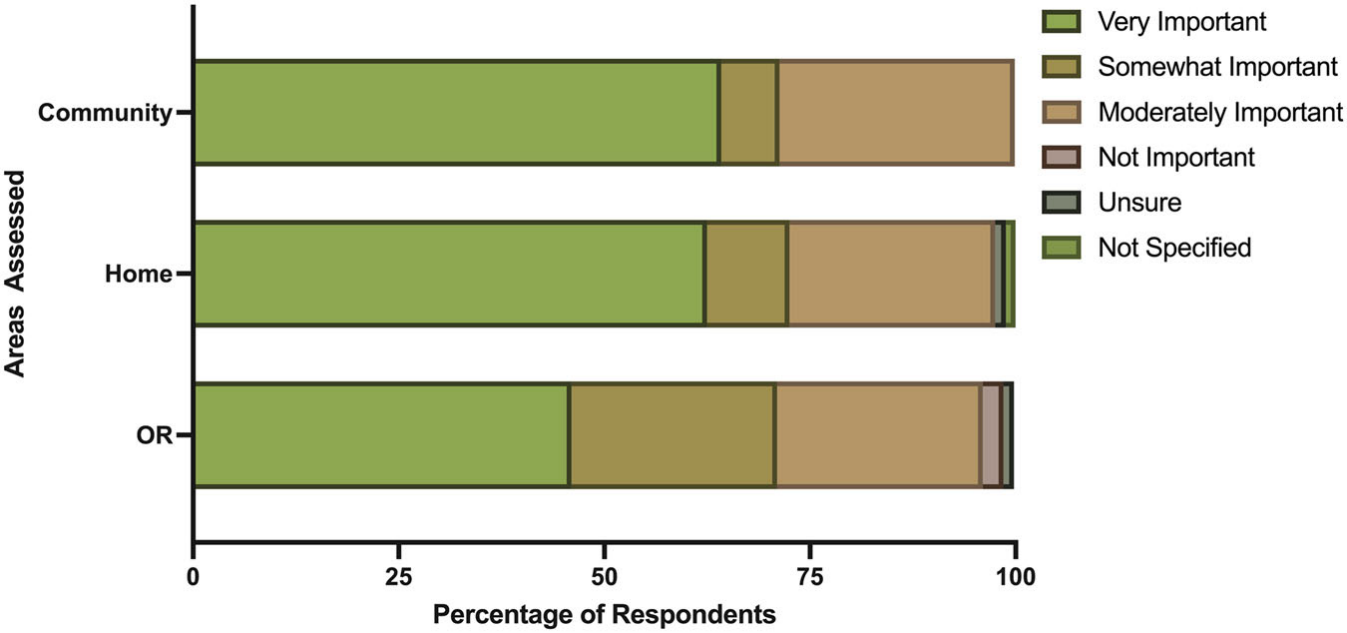
Importance of environmental sustainability in the following areas of the respondents' lives. OR, operating room.

Regarding the importance of environmental sustainability, most respondents agreed that it is very important at home (62%) and in their community (64%) (Figure 3). However, only 46% rated environmental sustainability as very important in the OR, 50% ranked it as moderately or somewhat important, and only 2% stated it is not important (Figure 3). There was no significant difference between attendings' and residents'/fellows' responses.

### 4 Institutional Practices

Regarding institutional practices, 51% of respondents felt that significant improvements in environmental sustainability could be made at their institution and 60% were very or somewhat unsatisfied with their institution's environmental sustainability practices, only 5% were very satisfied (Table 2). Most respondents reported that environmental initiatives at their institution were not occurring (24%), or they were unsure if they were occurring (46%), while 28% responded they had already begun or were upcoming (Table 2).

**Table 2:** Perceptions of Institutional Practices Regarding Environmental Sustainability in Operating Rooms.

Variable	Overall, n = 80, N (%)
Improvements can be made at my institution	
Significantly	41 (51.3)
Somewhat	30 (37.5)
Nothing can be done	1 (1.3)
Actively making efforts	3 (3.8)
Do not think it is an issue	1 (1.3)
Not specified	4 (5.0)
Satisfaction with institutional practices	
Very satisfied	4 (5.0)
Somewhat satisfied	13 (16.3)
Neutral	13 (16.3)
Somewhat unsatisfied	23 (28.8)
Very unsatisfied	25 (31.3)
Unsure	2 (2.5)
Environmental initiatives at my institution	
Already begun	11 (13.8)
Upcoming	11 (13.8)
Not occurring	19 (23.8)
Unsure	37 (46.3)
Not specified	2 (2.5)

**Figure 4:**
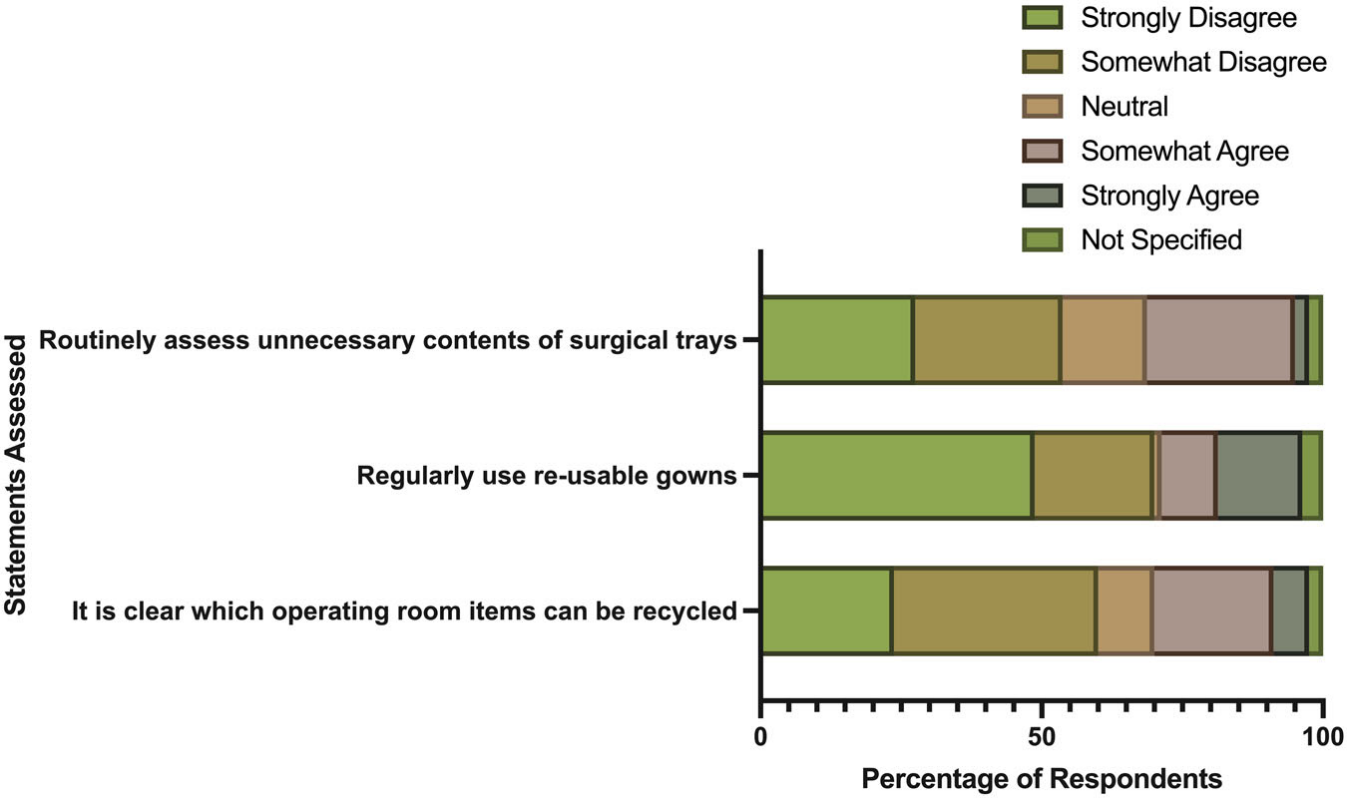
Respondents' institutional practices regarding environmental sustainability in operating rooms.

Only 6% of respondents strongly agreed that it was clear which OR items can be recycled, 21% somewhat agree, and 60% somewhat or strongly disagree (Figure 4). Most respondents strongly disagreed (49%) or somewhat disagreed (21%) that reusable gowns were routinely used (Figure 4). Only 2% of respondents strongly agreed that they routinely assessed unnecessary contents of surgical trays, 26% somewhat agreed, and 54% strongly or somewhat disagreed (Figure 4).

### 5 Barriers, Education, and Training

The most prevalent barriers to environmental sustainability in the OR were lack of incentives (68%), lack of hospital supports (60%), lack of information/knowledge (59%), cost (58%), lack of time (50%), and inconvenience (48%) (Table 3).

**Table 3:** Perceptions of Barriers and Educational Initiatives for Environmental Sustainability in Operating Rooms.

Variable	Overall, n = 80, N (%)
Barriers to environmental sustainability	
Lack of incentives	54 (67.5)
Lack of hospital support	48 (60.0)
Lack of information/knowledge	47 (58.8)
Cost	46 (57.5)
Lack of time	40 (50.0)
Inconvenience	38 (47.5)
Lack of resources/tools	31 (38.8)
Handling contaminated materials	21 (26.3)
Inconvenience	38 (47.5)
Staff attitudes	26 (32.5)
Reprocessing requirements	37 (46.3)
We simply do not	5 (6.3)
No barriers	1 (1.3)
Other	
Infection prevention control	1 (1.3)
Regulations	1 (1.3)
Commercial interests	1 (1.3)
Not specified	1 (1.3)
Have you received education on waste reduction and sustainability in the operating room?	
No	40 (50.0)
From peer‐to‐peer discussion	17 (21.3)
From independent reading	16 (20.0)
From conferences	14 (17.5)
From continuing education events	13 (16.3)
From journal club	8 (10.0)
From medical societies	7 (8.8)
From department	6 (7.5)
From hospital	5 (6.3)
From university	3 (3.8)
Other	
Residency	1 (1.3)
Interested colleagues	2 (2.5)
What do you think is a beneficial format for education on environmental sustainability?	
Small group workshops at individual hospitals	52 (65.0)
Conference lectures	38 (47.4)
Formal curriculum during medical school/residency	30 (37.5)
Online e‐modules	24 (30)
Other	
Education not the issue	2 (2.5)
Not specified	2 (2.5)

Of those involved in a residency program, 89% (n = 49/55) reported that their residency program provided no education on environmental sustainability, or they were unsure if it did (32%) (Table 4). Most strongly (39%) or somewhat agreed (28%) that medical trainees should be provided formal education on this topic (Table 4). Most respondents strongly (29%) or somewhat agreed (42%) that they would attend future educational events on this topic (Table 4). Overall, respondents were open to small group workshops (65%), conference lectures (47%), formal curriculum (38%), and online e‐modules (30%) (Table 3).

**Table 4:** Perceptions of Current and Future Formal Education on Environmental Sustainability in Operating Rooms.

Variable	Overall, n = 80, N (%)
How many hours does your residency curriculum dedicate to climate change and its impact on health?	
None	31 (38.8)
1‐5	6 (7.5)
6‐10	0
>10	0
Unsure	18 (22.5)
Not involved in a residency program	25 (31.3)
Do you think medical trainees should be provided formal education on environmental sustainability?	
Strongly agree	31 (38.8)
Somewhat agree	22 (27.5)
Neutral	14 (17.5)
Somewhat disagree	6 (7.6)
Strongly disagree	4 (5.0)
Not specified	3 (3.8)
Would you attend future educational events regarding environmental sustainability in healthcare?	
Strongly agree	23 (28.8)
Somewhat agree	34 (42.5)
Neutral	13 (16.3)
Somewhat disagree	5 (6.3)
Strongly disagree	2 (2.5)
Not specified	3 (3.8)

### 6 Qualitative Analysis

#### 6.1 Current Initiatives

Fourteen respondents identified that some form of sustainability initiative has been attempted in their practice. Initiatives reported among respondents included forming committees, introducing recycling programs, tray reviews, paperless charting, implementing reusable instruments, and reducing draping. However, approximately 21% of these respondents reported that these initiatives had been abandoned or are difficult to carry out.

#### 6.2 Additional Barriers and Future Directions

Respondents identified several additional barriers regarding environmental sustainability. Respondents indicated that in busy ORs, it is challenging to make environmental sustainability a priority. Furthermore, it was noted that hospital policy and processes were a hindrance, and that systemic change would be required. Subsequently, respondents felt a sense of futility and resistance when attempting to make such systemic change.

Respondents highlighted that future directions should include addressing equipment packaging/reprocessing, anesthetic gas use, and engaging in discussions of environmental sustainability with policy makers.

## Discussion

This is the first study surveying otolaryngologists on environmental sustainability in the OR. The results show that Canadian otolaryngologists believe in climate change and the impact of GHGs. They believe climate crisis will impact future generations; however, they are less certain about its effects on themselves, their patients, and their communities. Similarly, they recognize the importance of environmental sustainability in their personal lives, but there is less certainty regarding the contribution of ORs to the climate crisis.

Previous surveys have shown a high interest in increasing recycling in ORs by anesthesiologists (80%–97%).^25^ Similarly, a survey of academic surgeons in the United States revealed that 90% agreed or strongly agreed that waste of sterile surgical items is an issue.^26^ In our study, 46% of respondents ranked environmental sustainability as very important in the OR, which is less than those who ranked it as very important at home (62%) and in the community (64%). The lower ranking of importance in the OR may be due to variations in how the questions were posed; the previous studies asked about recycling and sterile waste in the OR, whereas this study asked about environmental sustainability overall. This may also represent a complacency with OR practices and their environmental impacts, or it may suggest a need for further education within OHNS on the environmental impacts of ORs.

A lack of information and knowledge was reported to be a barrier to environmental sustainability by 59% of respondents. Further, 89% of those involved in a residency program stated that their residency program provided no education on environmental sustainability or they were unsure if it did. This theme seems consistent across levels of medical education as Canadian medical students recently called for incorporation of “Planetary Health Educational Competencies” into medical education curriculums.^28^ In this survey, most respondents strongly or somewhat agreed that formal education on environmental sustainability should be developed (66%). Frameworks for implementing postgraduate eco‐education suggest highlighting the impacts of climate change on patient presentations and encouraging adaptation of current clinical practices.^29^, ^30^ This may be effective within otolaryngology, as fewer respondents in our study felt that climate change will significantly harm their patients (28%) compared to future generations (81%), suggesting a feeling of distance from its impacts. However, the climate crisis already impacts OHNS patients; it increases aeroallergens, worsens air quality, and increases ultraviolet radiation, subsequently increasing the prevalence of upper airway diseases and cutaneous malignancies of the head and neck.^31^, ^32^, ^33^, ^34^, ^35^, ^36^, ^37^, ^38^, ^39^ Improving education on the current impacts of climate change on OHNS patients may motivate eco‐action in otolaryngology practices accordingly.

Most otolaryngologists were not aware of which OR items could be recycled, did not use reusable gowns, and did not routinely assess unnecessary contents of surgical trays. These are examples of eco‐action which can be taken by the practicing otolaryngologists. Reviewing surgical tray contents can reduce unnecessary reprocessing/waste, costs, and set‐up time, while maintaining surgeon satisfaction.^31^, ^40^, ^41^, ^42^, ^43^, ^44^ Reusable gowns are shown to produce less GHGs than disposable gowns per 1000 uses.^45^ As noted in our thematic analyses, collaboration with anesthesia to consider total intravenous anesthesia (TIVA) or regional/neuraxial blocks can mitigate the environmental impact of anesthetic gases.^31^, ^46^, ^47^, ^48^ Ultimately, these changes require a top‐down approach beginning with systems level engagement, incentives, and mandates. However, buy‐in from staff is required to facilitate these changes and ensure they are effective. Currently there is still ambivalence from Canadian otolaryngologists regarding environmentally friendly impacts in the OR. Therefore, focusing on educational initiatives may be most appropriate. Our study found that 48% of attendings had not received education on environmental sustainability in the OR and implementing education at only the trainee level would miss this demographic. Continuing medical education events may look to include environmental topics. Ideally, small‐group educational sessions and committees or “green teams” fostering eco‐action could be developed at individual hospitals.

This survey is not without limitations. It is cross‐sectional and, therefore, only provides a snapshot in time. More survey questions may provide more insights into this topic; however, we attempted to make the survey short enough to promote participation. This survey yielded an 11% response rate. While higher response rates would be preferable, 11% is within the norms for previous surveys of the CSOHNS.^49^, ^50^ Nonetheless, the small sample size may have introduced a selection bias into the results, as those interested and concerned regarding environmental sustainability are more likely to respond. This may have skewed responses toward greater concern regarding the environment and therefore may not be fully representative of all Canadian Otolaryngologists. However, this further emphasizes the novelty of the results, if those most invested in environmental sustainability responded to this survey; then it is surprising that there was ambivalence regarding the impact of ORs on the environment.

## Conclusion

This study provides valuable insights into Canadian otolaryngologists' attitudes, beliefs, practice patterns, and educational experiences regarding environmental sustainability in ORs. The results highlight a current level of ambivalence toward environmental sustainability in ORs which may be remedied with further education leading to systemic changes and reduction of barriers, ultimately resulting in the improvement of environmental sustainability in OHNS ORs.

## Author Contributions

**Kalpesh Hathi**, study design, survey development, interpreting data, writing manuscript; **James Fowler**, study design, survey development, interpreting data, revising manuscript, supervision; **Sarah Zahabi**, survey development, survey translation, interpreting data, revising manuscript; **Agnieszka Dzioba**, study design, survey development, data analysis planning, interpreting data, submitting and revising REB application, revising manuscript; **Edward Madou**, data analysis, interpreting data, revising manuscript; **Anna C. Gunz**, study design, survey development, interpreting data, revising manuscript; **Leigh J. Sowerby**, study design, survey testing and revision, interpreting data, revising manuscript; **Anthony C. Nichols**, study design, survey testing and revision, interpreting data, revising manuscript; **Julie E. Strychowsky**, study initiation and conceptualization, study design, survey development, interpreting data, revising manuscript, supervision.

## Disclosures

### 1 Competing interests

The authors declare no potential conflicts of interest in the past 24 months.

### 2 Funding source

None.

## Supporting information

supporting Information

supporting Information
