# Supplementary material for: Attitudes and Perceptions of Canadian Otolaryngology‐Head and Neck Surgeons and Residents on Environmental Sustainability
Source: OTO Open. 2023 Feb 23;7(1):e40. doi: 10.1002/oto2.40 (PMC10046710; doi:10.1002/oto2.40)
Supplement: Supplementary file 1 — supporting Information [file OTO2-7-e40-s002.docx]

**Supplemental Material 1**

**SECTION 1: DEMOGRAPHICS**

1. What is your position?
   1. Resident
   2. Fellow
   3. Attending
2. How many years have you been in practice? [If attending]
   1. <10 years
   2. 10-19 years
   3. 20-29 years
   4. 30+ years
3. What best describes your main practice scope(s)? (Select all that apply) [If attending; or If fellow]
   1. General Otolaryngology-Head and Neck Surgery
   2. Pediatric Otolaryngology-Head and Neck Surgery
   3. Rhinology/Skull Base Surgery
   4. Laryngology
   5. Otology/Neurotology
   6. Head and Neck Oncology
   7. Facial Plastics and Reconstructive Surgery
   8. Sleep Surgery
4. How old are you?
   1. 20-29
   2. 30-39
   3. 40-49
   4. 50-59
   5. 60-69
   6. 70+
5. What is your identified gender? (Select all that apply)
6. Female/Woman
7. Male/Man
8. Transgender
9. Non-binary or gender-fluid
10. Two-spirit
11. Prefer not to answer.
12. Prefer to self-identify: ______________
13. What is your province or territory of practice?
    1. Alberta
    2. British Columbia
    3. Manitoba
    4. New Brunswick
    5. Newfoundland and Labrador
    6. Northwest Territories
    7. Nova Scotia
    8. Nunavut
    9. Ontario
    10. Prince Edward Island
    11. Quebec
    12. Saskatchewan
    13. Yukon
14. What best describes your practice setting?
    1. Isolated/Remote community practice
    2. Rural community practice
    3. Urban community practice
    4. Academic practice

**SECTION 2: GENERAL CLIMATE BELIEFS AND ATTITUDES**

1. Do you believe that climate change is occurring? Climate change refers to the concept that the world’s average temperature has been increasing for the past 50 to 100 years, and, if greenhouse gas emissions are not reduced, will continue to increase in the future, which will change the world’s climate.
2. Yes, I strongly believe in climate change
3. Yes, I somewhat believe in climate change
4. I am unsure about climate change
5. No, I do not believe climate change is occurring
6. How much do you think climate change will harm the following?

|  | Not at all (1) | Somewhat (2) | Moderately (3) | Significantly (4) | Unsure (5) |
| --- | --- | --- | --- | --- | --- |
| You personally (1) |  |  |  |  |  |
| People in your community (2) |  |  |  |  |  |
| Your patients (3) |  |  |  |  |  |
| People in your country (4) |  |  |  |  |  |
| Future generations (5) |  |  |  |  |  |

**SECTION 3: OPERATING ROOM ENVIRONMENTAL SUSTAINABILITY ATTITUDES**

1. How strongly do you agree with the following statement?

|  | Not important at all (1) | Somewhat important (2) | Moderately important (3) | Very important (4) | Unsure (5) |
| --- | --- | --- | --- | --- | --- |
| The operating room (1) |  |  |  |  |  |
| At home (2) |  |  |  |  |  |
| In the community (3) |  |  |  |  |  |

|  | Strongly Disagree (1) | Somewhat Disagree (2) | Unsure (3) | Somewhat Agree (4) | Strongly Agree (5) |
| --- | --- | --- | --- | --- | --- |
| Operating room waste is a significant contributor to the global environmental crisis |  |  |  |  |  |

1. How important is improving environmental sustainability to you in the following areas?
2. Do you believe significant improvements could be made at your institution regarding operating room waste and environmentally friendly practices (e.g., using re-usable tools and surgical gowns, reducing unnecessary surgical drapes, recycling, etc.)?
   1. Yes, significant improvements could be made
   2. Yes, some improvements could be made
   3. No, it is an issue, but there is nothing further that could be done at this time
   4. No, we actively make efforts in this domain
   5. No, I do not think it is an issue

**SECTION 4: PRACTICE PATTERNS/BARRIERS**

1. How satisfied are you with the environmentally friendly practices at your institution?
   1. Very satisfied
   2. Somewhat satisfied
   3. Unsure
   4. Somewhat unsatisfied
   5. Very unsatisfied
2. How strongly do you agree with the following statements about your institution?

|  | Strongly Disagree (1) | Somewhat Disagree (2) | Unsure (3) | Somewhat Agree (4) | Strongly Agree (5) |
| --- | --- | --- | --- | --- | --- |
| It is clear which operating room items can be recycled (1) |  |  |  |  |  |
| We regularly use re-usable gowns for procedures (2) |  |  |  |  |  |
| We routinely assess unnecessary contents of surgical trays (3) |  |  |  |  |  |

1. Are there plans/initiatives to improve environmental sustainability at your hospital/department?
   1. Yes, we have already begun
   2. Yes, upcoming
   3. No
   4. Unsure
2. Which of the following do you perceive are the biggest barriers to increasing environmental sustainability in the operating room? (Select all that apply)
   1. Cost
   2. Lack of resources and tools
   3. Lack of information/knowledge
   4. Lack of time
   5. Lack of incentive
   6. Handling of contaminated materials
   7. It is inconvenient
   8. Lack of support from hospital/leadership
   9. Staff attitudes
   10. Reprocessing requirements
   11. We simply do not
   12. No perceived barriers
   13. Other, please specify:

**SECTION 5: TRAINING & EDUCATION**

1. Have you previously received education regarding reducing waste and increasing environmental sustainability in the operating room? (Select all that apply)
   1. No
   2. Yes, from medical societies
   3. Yes, from my department
   4. Yes, from my hospital
   5. Yes, from my university
   6. Yes, from continuing medical education events
   7. Yes, from conferences
   8. Yes, from peer-to-peer discussion
   9. Yes, from journal club
   10. Yes, from independent reading
   11. Yes, from other, please specify:
2. What would be the best format to deliver operating room focused environmental sustainability education?
   1. Small group workshops at individual hospitals
   2. Formal curriculum during medical school and residency/fellowship
   3. Online e-modules
   4. Conference lectures
   5. Other, please specify:
3. Do you teach medical students/residents/fellows, who rotate with/shadow you about environmental sustainability in healthcare?
   1. Yes
   2. Sometimes
   3. No
4. How strongly do you agree with the following statements?

|  | Strongly Disagree (1) | Somewhat Disagree (2) | Unsure (3) | Somewhat Agree (4) | Strongly Agree (5) |
| --- | --- | --- | --- | --- | --- |
| Medical trainees should be provided with formal teaching regarding environmental sustainability and healthcare (1) |  |  |  |  |  |
| I would attend future educational events regarding environmental sustainability in healthcare (2) |  |  |  |  |  |

1. Are you involved in a residency training program? [If attending]
   1. Yes
   2. No
2. How many hours does your residency curriculum currently dedicate to covering the topic of climate change and its potential impact on health? [If resident; If attending involved in a residency training program]
   1. None
   2. 1-5
   3. 6-10
   4. >10
   5. Unsure

**SECTION 6: OPEN-ENDED QUESTIONS**

1. Please describe any environmental sustainability initiatives you and/or your colleagues have started:
2. Please provide any further comments regarding barriers, future directions, or personal feelings regarding environmental sustainability:
