# Supplementary material for: Attitudes and Perceptions of Canadian Otolaryngology‐Head and Neck Surgeons and Residents on Environmental Sustainability
Source: OTO Open. 2023 Feb 23;7(1):e40. doi: 10.1002/oto2.40 (PMC10046710; doi:10.1002/oto2.40)
Supplement: Supplementary file 2 — supporting Information [file OTO2-7-e40-s001.docx]

**Supplemental Material 2**

**SÉCTION 1: CHARACTÉRISTIQUES DÉMOGRAPHIQUES**

1. Quel est votre titre?
   1. Résident
   2. Fellow
   3. Patron
2. Si vous êtes patron, depuis combien d’années êtes-vous en pratique?
   1. <10 années
   2. 10-19 années
   3. 20-29 années
   4. 30+ années
3. Lequel des options suivantes décrit le mieux votre pratique? (Sélectionner tout option applicable).
   1. Otorhinolaryngologie générale
   2. Otorhinolaryngologie pédiatrique
   3. Rhinologie/Base du crâne
   4. Laryngologie
   5. Otologie/Neurotologie
   6. Oncologie cervico-faciale
   7. Chirurgie plastique faciale
4. Quel âge avez-vous?
   1. 20-29 ans
   2. 30-39 ans
   3. 40-49 ans
   4. 50-59 ans
   5. 60-69 ans
   6. 70+ ans
5. À quel genre vous identifiez-vous? (Sélectionner tout option applicable)
6. Femme
7. Homme
8. Transgenre
9. Non-binaire
10. Bispirituel
11. Je préfère ne pas répondre.
12. Je préfère m’identifier comme: ______________
13. Où pratiquez-vous?
    1. Alberta
    2. Colombie-Britannique
    3. Manitoba
    4. Nouveau-Brunswick
    5. Terre-Neuve et Labrador
    6. Territoires du nord-ouest
    7. Nouvelle-Écosse
    8. Nunavut
    9. Ontario
    10. L’Île-du-Prince-Édouard
    11. Québec
    12. Saskatchewan
    13. Yukon
14. Lequel des éléments suivants décrit le mieux votre lieu de pratique?
    1. Pratique en communauté isolé
    2. Pratique en communauté rurale
    3. Pratique en communauté urbaine
    4. Pratique académique

**SECTION 2: CROYANCES ET ATTITUDES ENVERS LE CHANGEMENT CLIMATIQUE**

1. Croyez-vous que le changement climatique se produit? Le changement climatique est le concept selon lequel la température moyenne mondiale augmente depuis 50 à 100 ans, et qui, si les émissions de gaz à l’effet de serre ne sont pas réduites, continuera d’augmenter en ayant des conséquences sur le climat mondial.
2. Oui, j’y croit fortement
3. Oui, j’y croit
4. Je suis incertain (e)
5. Non, je ne crois pas que le changement climatique se produit
6. À quel point est-ce que vous jugez que le changement climatique nuirait aux items suivants ?

|  | Pas du tout (1) | Un peu (2) | Modérément (3) | De façon significative (4) | Incertain (5) |
| --- | --- | --- | --- | --- | --- |
| Toi personnellement (1) |  |  |  |  |  |
| Les gens dans votre communauté (2) |  |  |  |  |  |
| Vos patients (3) |  |  |  |  |  |
| Les personnes dans votre pays (4) |  |  |  |  |  |
| Les futures générations (5) |  |  |  |  |  |
|  |  |  |  |  |  |

**SECTION 3: ATTITUDES ENVERS LA DURABILITÉ ENVIRONNEMENTALE EN SALLE OPÉRATOIRE**

|  | Tout à fait en désaccord (1) | En désaccord (2) | Incertain (3) | En accord (4) | Tout à fait en accord (5) |
| --- | --- | --- | --- | --- | --- |
| Les déchets en salle opératoire est un contribuant significatif à la crise globale de l’environnement |  |  |  |  |  |

1. À quel point êtes-vous en accord avec les items suivants?
2. À quel point est-il important d’améliorer la durabilité environnementale dans les domaines suivants?

|  | Pas du tout important (1) | Peu important (2) | Relativement important (3) | Très important (4) | Incertain (5) |
| --- | --- | --- | --- | --- | --- |
| La salle d’opération (1) |  |  |  |  |  |
| À la maison (2) |  |  |  |  |  |
| Dans la communauté (3) |  |  |  |  |  |

1. Croyez-vous que des améliorations significatives pourraient être apportées dans votre institution en ce qui concerne les déchets en salle opératoire et les pratiques liées à la durabilité environnementale (par exemple, outils et blouses chirurgicales réutilisables, minimisation d’utilisation de draps chirurgicaux non-nécessaires, le recyclage, etc.)?
   1. Oui, des améliorations importantes pourraient être apportées
   2. Oui, quelques améliorations pourraient être apportées
   3. Non, c’est un enjeu, mais il n’y a rien qui pourrait être fait en ce moment pour améliorer la situation
   4. Non, on fait déjà des efforts dance ce domaine
   5. Non, je ne pense pas que c’est un enjeu

**SECTION 4: TENDANCES/BARRIÈRES DE PRATIQUE**

1. Dans quelle mesure êtes-vous satisfait des pratiques de durabilité environnementale dans votre institution de pratique ?
   1. Très satisfait
   2. Satisfait
   3. Incertain
   4. Peu satisfait
   5. Pas satisfait
2. Dans quelle mesure êtes-vous d'accord avec les items suivants au sujet de votre institution de pratique ?

|  | Tout à fait en désaccord (1) | En désaccord (2) | Incertain (3) | En accord (4) | Tout à fait en accord (5) |
| --- | --- | --- | --- | --- | --- |
| Les articles dans la salle d’opération qui peuvent être recyclés sont évidents (1) |  |  |  |  |  |
| On utilise régulièrement des blouses chirurgicales réutilisables pour les procédures (2) |  |  |  |  |  |
| On évalue de façon routinière les items non-nécessaires inclut dans les plateaux chirurgicales (3) |  |  |  |  |  |

1. Existe-t-il des plans ou initiatives pour améliorer la durabilité environnementale dans votre hôpital/département?
   1. Oui, nous avons déjà initié
   2. Oui, bientôt
   3. Non
   4. Incertain
2. Lesquels des éléments suivants considérez-vous comme le plus grand obstacle à l’augmentation de la durabilité de l’environnement dans la salle opératoire? **(Sélectionner 3)**
   1. Manque de ressources et outils
   2. Manque d’informations et connaissances
   3. Manque de temps
   4. Manque d’incitations
   5. Manutention de matériaux contaminés
   6. C’est inconvénient
   7. Manque de support de la part de l’hôpital/l’administration
   8. Attitudes des employés
   9. On en fait pas
   10. Pas de barrières
   11. Autre, veuillez spécifier :

**SECTION 5: FORMATION ET EDUCATION**

1. Avez-vous déjà reçu une formation formelle sur le sujet de la durabilité environnementale et la réduction de déchets dans la salle opératoire? (Veuillez choisir tous ceux qui s’appliquent)
   1. Non
   2. Oui, de la part des sociétés médicales
   3. Oui, de la part de mon département
   4. Oui, de la part de mon hôpital
   5. Oui, de la part de mon université
   6. Oui, en participant à des évènements de formation médicale continue
   7. Oui, en assistant à des conférences
   8. Oui, en participant à des discussions entre pairs
   9. Oui, en participant à des « journal club »
   10. Oui, en faisant de la lecture indépendante
   11. Oui, d’autre source, veuillez spécifier :
2. Quel serait le meilleur format pour enseigner la durabilité environnementale dans la salle opératoire?
   1. Ateliers petits groups dans les hôpitaux individuels
   2. Curriculum formel pendant les études en médecines ou la résidence/fellowship
   3. Des modules de formation en ligne
   4. Conférences ou cours
   5. Autre, veuillez spécifier :
3. Enseignez-vous les étudiants en médecine/résidents/fellows, avec qui vous travaillez, au sujet de la durabilité environnementale et les soins de santé?
   1. Oui
   2. Quelquefois
   3. Non
4. Êtes-vous en accord ou désaccord avec les énoncés suivants :

|  | Tout à fait en désaccord (1) | En désaccord (2) | Incertain (3) | En accord (4) | Tout à fait en accord (5) |
| --- | --- | --- | --- | --- | --- |
| Stagiaires en médecine devraient recevoir une formation au sujet de la durabilité environnementale et les soins de santé (1) |  |  |  |  |  |
| J’assisterais aux événements éducationnels au sujet de la durabilité environnementale et les soins de santé (2) |  |  |  |  |  |

1. Participez-vous à un programme de résidence en tant que superviseur de résidents ou en tant que participant à l’enseignement des résidents ou l’administration du programme ? [Si patron]
   1. Oui
   2. Non

**SECTION 6: QUESTIONS OUVERTES**

1. Veuillez s’il vous plait décrire toute initiative de durabilité environnementale que vous ou vos collègues avez initié :
2. S’il vous plait veuillez inclure tout commentaire sur les obstacles, orientations futures ou sentiments personnels au sujet de la durabilité environnementale :
